# Dietary chicory rhamnogalacturonan-I modulates gut microbiota and immune responses in healthy adults

**DOI:** 10.20517/mrr.2026.04

**Published:** 2026-05-13

**Authors:** Evangelia N. Kerezoudi, Sue McKay, Seta Kurt, Maaike De Kreek, Jelle De Medts, Lynn Verstrepen, Jonas Ghyselinck, Lieven Van Meulebroek, Wim Calame, Ruud Albers, Annick Mercenier, Robert J. Brummer, Ignacio Rangel

**Affiliations:** ^1^School of Medical Sciences, Örebro University, Örebro 70182, Sweden.; ^2^Nutrition-Gut-Brain Interactions Research Centre, Food and Health Center, Örebro University, Örebro 70182, Sweden.; ^3^NutriLeads BV, Wageningen 6708 WH, The Netherlands.; ^4^Department of Clinical Research Laboratory, Faculty of Medicine and Health, School of Medical Sciences, Örebro University, Örebro 70182, Sweden.; ^5^Clinical and Experimental Endocrinology, KU Leuven, Leuven 3000, Belgium.; ^6^ProDigest BV, Zwijnaarde 9052, Belgium.; ^7^Laboratory of Integrative Metabolomics, Department of Translational Physiology, Infectiology and Public Health, Faculty of Veterinary Medicine, Ghent University, Merelbeke 9820, Belgium.; ^8^StatistiCal BV, Wassenaar 2241 MN, The Netherlands.

**Keywords:** Rhamnogalacturonan-I, chicory root fiber, prebiotic, bifidobacteria, gut-immune axis, randomized controlled trial

## Abstract

**Background:** Pectic rhamnogalacturonan-I (RG-I) is a dietary fiber that modulates the gut-immune axis. This study evaluates a novel variant of RG-I from chicory root (chRG-I).

**Methods:** In a randomized, double-blind, placebo-controlled trial, 55 healthy adults were stratified by habitual fiber intake and baseline *Bifidobacterium* levels before receiving 500 mg/day of chRG-I or placebo for four weeks. Primary endpoints included fecal *Bifidobacterium* counts. Secondary outcomes assessed fecal metabolites, systemic immune cell activation markers, and gastrointestinal symptoms. To provide mechanistic insights, donor-matched fecal samples were used in *in vitro* fermentation and Caco-2/peripheral blood mononuclear cell co-culture gut barrier models.

**Results:** Supplementation with chRG-I induced a statistically significant bifidogenic effect, with absolute levels peaking at week three, and lower levels of some fecal short-chain fatty acids (SCFA) compared to placebo. However, donor-matched *in vitro* fermentations with chRG-I confirmed robust production of SCFA and reduction of branched-chain fatty acids levels (BCFA). Systemically, chRG-I upregulated HLA-DR expression on myeloid dendritic cells. Clinically, chRG-I was well-tolerated and slightly improved stool consistency compared to placebo. In an intestinal barrier challenge model, chRG-I fermentates (a pool of metabolites including SCFA and fragments of chRG-I) protected barrier integrity, modulated the cytokine milieu away from a predominantly pro-inflammatory response, as characterized by increased IL4 and IL22 and reduced IL9, IL17A, and IL21.

**Conclusion:** Supplementation with a low dose of chRG-I is well-tolerated, beneficially modulates the gut microbiome - which can protect the intestinal barrier, and subtly enhances systemic immune readiness, suggesting that chRG-I may have benefits as a functional food ingredient.

## INTRODUCTION

Gut health is the basis for overall well-being and can be affected by several factors including the host diet as well as the composition and function of the resident gut microbiota. A healthy gut microbiota can strengthen the gut barrier and provide protection from pathogens, as well as influence immunity, metabolism and even mental health via the production of signaling molecules such as short chain fatty acids (SCFA) and other microbial metabolites^[1]^. High diversity of the gut microbiota has been associated with a reduced risk of developing non-communicable diseases, and a varied diet with an assortment of different fibers can support gut microbiota diversity.

Dietary fibers derived from pectic polysaccharides are increasingly recognized for their ability to influence intestinal and immune functions through mechanisms that extend beyond microbial fermentation^[2,3]^. Among these, rhamnogalacturonan-I (RG-I) represents a structurally complex and biologically active domain of pectin, consisting of a backbone of (→ 2)-α-L-Rhap-(1 → 4)-α-D-GalpA-(1 →) units variably substituted with arabinan, galactan and arabinogalactan side chains^[4]^. The molecular composition of RG-I depends on its botanical origin^[5]^, but also on the plant tissue, developmental stage, and extraction parameters^[6]^ that all influence side-chain topology and esterification patterns, leading to a structural heterogeneity that may translate into functional diversity^[7]^. These structural features have been reported to influence physiological effects e.g., interaction with immune cells^[8,9]^, as well the gut microbiota composition and function^[10]^.

RG-I derived from chicory root (chRG-I) is characterized by distinct molecular features, including its molecular weight distribution profile, degree of acetylation and methyl-esterification, and side-chain architecture. Increasing evidence indicates that such structural attributes, particularly branching patterns and side-chain composition, can influence biological activity, including immune-modulatory effects. In line with this, recent work on pectic polysaccharides from alternative plant sources, such as *Gardenia jasminoides*, has demonstrated that structural features of RG-I-rich domains are closely linked to immunomodulatory activity^[11]^. These observations support the notion that source- and structure-dependent differences in RG-I may contribute to differential biological responses. Furthermore, chicory root (*Cichorium intybus*) is globally processed for inulin extraction, generating substantial pomace that remains under-utilized despite its content of pectic polysaccharides, including RG-I^[12]^. Valorizing this side-stream aligns with circular-economy goals and may provide an additional source of functional dietary fiber.

Recent human intervention studies have shown that relatively low doses of RG-I (300 mg and 1,500 mg/day) can modulate gut microbiota composition in healthy adults, including promoting the growth of beneficial microbes such as bifidobacteria and influencing immune responsiveness^[13,14]^. Building on this foundation, the present study investigated the effects of 500 mg/day chRG-I, a structurally related but distinct fiber source, extending this line of human RG-I research to a novel fiber source within the same human intervention setting^[13]^. We hypothesized that dietary supplementation with chRG-I would modulate gut microbiota composition and function, and also display an immune modulatory effect. The primary objective of this randomized controlled intervention trial was to determine if dietary supplementation with chRG-I could stimulate the growth of *Bifidobacterium* spp. in healthy adults. Secondary outcomes included the determination of fecal SCFA and calprotectin (F_cal) levels and expression of activation markers on circulating immune cells. In addition, validated questionnaires were employed to explore effects on wellbeing and gastrointestinal tolerance. Complementary *in vitro* models (microbial fermentation and intestinal barrier integrity) were used to gain insights into how this fiber interacts with the gut environment. This work represents the first human intervention trial evaluating the prebiotic potential of chRG-I.

## METHODS

### Human dietary intervention

#### Study design and allocation procedure

**Figure 1:**
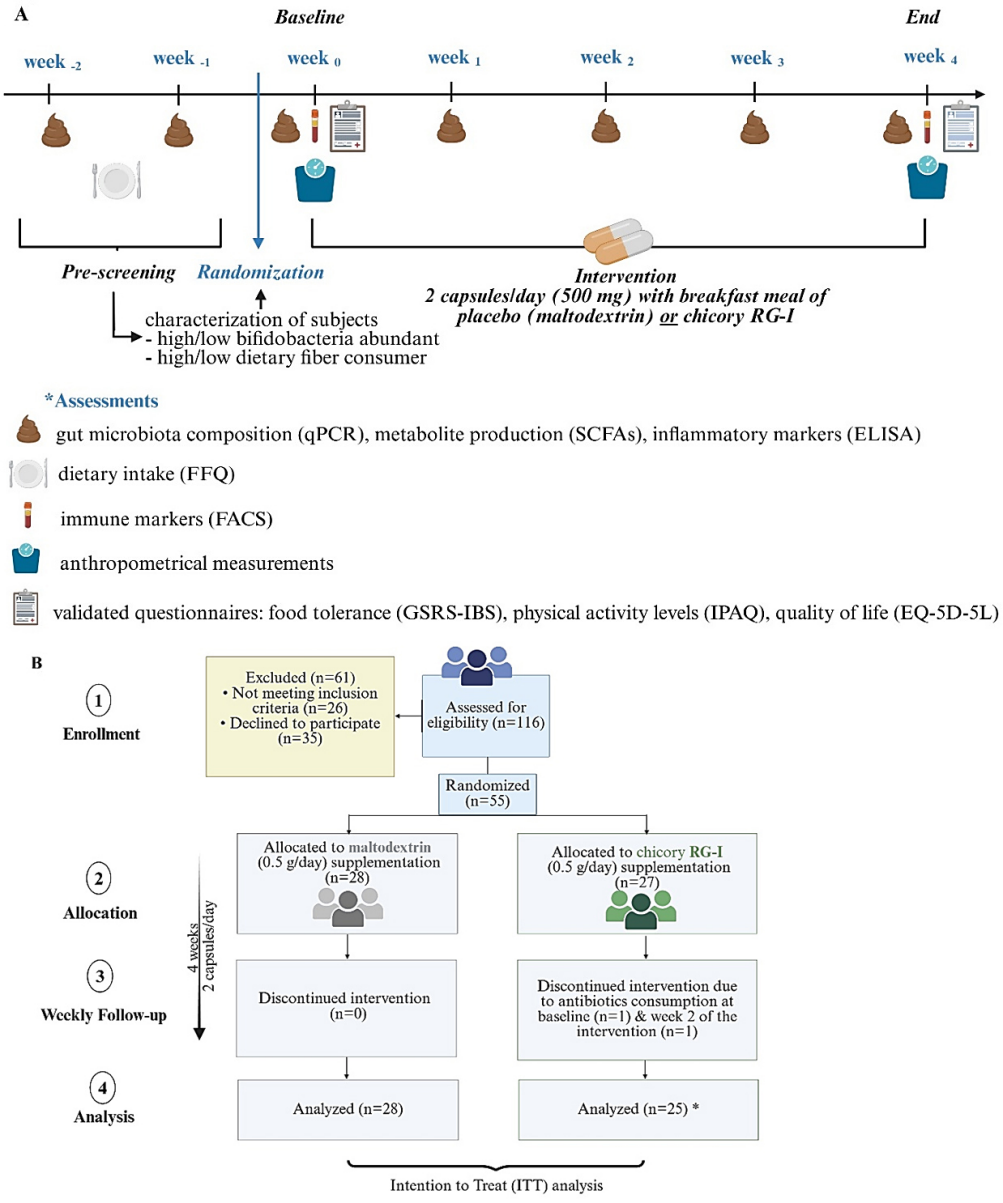
(A) Study design and (B) subjects’ disposition. *Participant numbers reflect those contributing data at each timepoint; at baseline, 27 individuals were included in the chRG-I group and 28 in the placebo group. (A) Created in BioRender. Kerezoudi, E. (2026). Available from: https://biorender.com/fs2dqxp; (B) Created in BioRender. Kerezoudi, E. (2026). Available from: https://biorender.com/2i6zeeh. chRG-I: RG-I derived from chicory root.

The study design, cohort organization, and detailed operational procedures [Figure 1A and B] have been described previously^[13]^. In brief, the study was set up with 3 arms to enable the use of one placebo group in comparison with either cRG-I^[13]^ or chRG-I (this paper). During a two-week run-in phase, baseline data on habitual dietary fiber intake and fecal *Bifidobacterium* spp. levels were collected. Participants that met the eligibility criteria were stratified as high/low fiber consumers (≥ or < 22.66 g/day) and as high or low *Bifidobacterium* carriers (≥ or < 0.894 log copies/µL, normalized to 100 ng/µL DNA), based on median values established in the first recruitment cohort (*n* = 20), in the absence of predefined biological cut-offs, and subsequently applied to participants enrolled later. This stratification approach was implemented to account for inter-individual variability in dietary habits and gut microbiota composition, both of which are known to influence responsiveness to dietary fiber interventions, and to ensure balanced group allocation across these factors^[15]^. Stratification was conducted by a researcher blinded to group allocation to optimize randomization across host- and microbiota-related variables.

A computer-generated randomization algorithm then assigned participants to receive either chRG-I (500 mg/day) or placebo (maltodextrin; 500 mg/day) for four weeks. Randomization and supplement coding were managed independently to ensure complete blinding of both participants and investigators until data analysis was completed. Participants were requested to take two capsules daily at breakfast while maintaining their habitual diet and lifestyle. Compliance was verified through capsule counts at the end of the intervention. All participants provided written informed consent prior to enrolment.

The study was performed according to Good Clinical Practice standards and met the ethical conditions of the Declaration of Helsinki. All participants signed informed consent documents describing the study’s aims and procedures. The study (Dnr. 2023-03938-01) was approved by the Swedish Ethical Review Authority and on 20 September 2023 it was registered https://clinicaltrials.gov/study/NCT06081972?term=NCT06081972&rank=1 (ID: NCT06081972).

#### Dietary intervention products

Chicory RG-I is a natural extract from the root of chicory. This patented, food grade ingredient, provided by NutriLeads (Wageningen, The Netherlands), is a water-soluble fermentable fiber enriched (> 90%) with the pectin RG-I. The monosaccharide composition of the chRG-I is shown in Table 1. The placebo consisted of maltodextrin (DE 15.8), a rapidly absorbed carbohydrate that is completely digested in the small intestine and therefore does not enter the colon or influence the colonic microbiome. This choice allowed the effects observed in the intervention group, relative to placebo, to be attributed to the presence of chRG-I. The test articles were made by Lab-medisan (Heerenveen, The Netherlands) as hydroxyproplymethycellulose (HMPC) capsules containing 0 g or 250 mg chRG-I-extract with maltodextrin as filler (MALDEX 170, Tereos, Aalst, Belgium) and with sunflower oil (0-10 mg) to support encapsulation. Test articles were packed as 15 capsules per blister strip, with 4 strips per pack. The test articles were identical for all treatments in capsule size, weight and color, as well as outer packaging.

**Table 1:** Monosaccharide composition of chRG-I

**Monosaccharides % (mol/mol)**									**Ratios and regions**			
	Fuc	Ara	Rha	Gal	Glc	Xyl	Man	GalA	GalA/Rha^1^	RG-I^2^	RG-I^3^	Ara/ Rha^4^
chRG-I	0.47± 0.06	56.4± 2.37	10.97± 0.25	12.7± 0.70	0.5± 0.17	0.43± 0.06	0.23± 0.06	18.27± 1.30	1.67± 0.12	91.1± 1.44	21.9± 1.44	5.17± 0.29

^1^Low GalA/Rha ratio indicates enrichment for RG‐I backbone; ^2^RG‐I domain calculated as (2*Rha + Ara + Gal); ^3^RG‐I backbone calculated as 2*Rha; ^4^Ara/Rha ratio indicates level of neutral Ara comprising sidechains linked to Rha. Fuc: fucose; Ara: arabinose; Rha: rhamnose; Gal: galactose; Glc: glucose; Xyl: xylose; Man: mannose; GalA: galacturonic acid; RG-I: rhamnogalacturonan-I domain. Data is shown as mean of three chRG-I batches ± standard deviation.

#### Procedures

Participants were recruited between September 2023 and March 2024 as part of a randomized controlled intervention study in healthy adults. Key eligibility criteria included adult individuals with no diagnosed chronic gastrointestinal or metabolic disorders and no recent use of antibiotics or microbiota-modulating supplements. Detailed inclusion and exclusion criteria are described elsewhere^[13]^. Biological samples were collected at predefined timepoints, including baseline and after four weeks of supplementation, with stool samples collected on a weekly basis throughout the intervention.

All procedures summarized below were conducted according to a previously established clinical protocol^[13]^. The same methodological framework covering participant recruitment, sampling, and analytical workflows, was applied to maintain procedural consistency across cohorts.

At baseline and after four weeks of supplementation, body height and weight were measured using calibrated instruments to determine body mass index (BMI). Stool samples were collected weekly using validated home-sampling kits, transported under cooled conditions, and stored at -80 °C until processing. Quantification of *Bifidobacterium* spp. was performed by quantitative PCR (qPCR) following previously described and validated methods^[13]^, and data was expressed as log *Bifidobacterium* copies per μL of extracted DNA, normalized to 100 ng/μL DNA (absolute numerical levels). Fecal SCFA (acetate, propionate, butyrate) and BCFA (isobutyrate, isovalerate, isocaproate) were assessed by gas chromatography, while F_cal concentrations were analyzed using a chemiluminescent immunoassay^[16]^.

Fasting venous blood samples were collected at baseline and post-intervention for isolation of peripheral blood mononuclear cells (PBMCs). Flow cytometric phenotyping was employed to determine the relative abundance and activation status of plasmacytoid dendritic cells (pDCs), myeloid dendritic cells (mDCs), and monocytes. Expression of activation markers, including CD86 and HLA-DR, was quantified as geometric mean fluorescence intensity (MFI) values. Fluorescence-minus-one (FMO) controls (CD86, CD304) and reference PBMC aliquots from a single donor were included in each run to ensure analytical precision and inter-assay reproducibility^[13]^.

Validated questionnaires were used to assess participants’ gastrointestinal symptoms, physical activity, quality of life and dietary habits. Dietary fiber intake was estimated using a computed Food Frequency Questionnaire (FFQ), an algorithm-based tool developed and validated at Örebro University^[17]^ to assess population-specific intake patterns and stratify participants according to habitual fiber consumption. The 15-item Gastrointestinal Symptom Rating Scale (GSRS) was used to evaluate five symptom domains i.e. diarrhea, constipation, abdominal pain, indigestion and reflux, on a seven-point Likert scale^[18]^. Physical activity was assessed using the International Physical Activity Questionnaire-Short Form (IPAQ-SF)^[19]^, and health-related quality of life was measured using the EQ-5D-5L (EuroQol Research Foundation), which also included a visual analogue scale (VAS) from 0 (“worst imaginable health”) to 100 (“best imaginable health”)^[20]^.

Further details on laboratory validation, data acquisition, and analytical procedures are available in an earlier publication^[13]^.

#### Outcomes

The primary outcomes comprised changes in the absolute counts of *Bifidobacterium* spp. and the proportion of participants exhibiting an increase in bifidobacteria counts. Demonstration of statistically significant effects of chRG-I on these parameters was regarded as evidence of efficacy. Any analyses outside the predefined primary outcomes were classified as exploratory.

### Preclinical biological function assays

#### Short-term in vitro batch fermentation (SCFA, BCFA, pH)

For the short-term colonic incubation period, reactors (70 mL volume) were filled with ProDigest’s SHIME® nutritional medium (PD01), representative of the colon environment. Prior to use, the medium was rendered anaerobic by boiling to remove oxygen.

chRG-I was then added to single reactors at a final concentration of 3.0 g/L. Blank control did not receive any test product (no-substrate control). Cryopreserved fecal suspensions (7.5% w/v; collected from *n* = 15 randomly selected volunteers in the chRG-I group at baseline, i.e., prior to dietary intervention) were added at 10% v/v, serving as the microbial inoculum. Incubations were carried out under anaerobic conditions at 37 °C for 48 h with mild shaking (90 rpm). Microbial metabolic activity was assessed at 0, 6, 24, and 48 h as described earlier^[21]^. chRG-I-supplemented incubations were compared with blank controls for changes in pH, and changes in concentrations of SCFA and BCFA.

#### Caco-2/PBMC co-culture assay with pokeweed mitogen (PWM) challenge (cytokines and TEER)

Supernatants from the short-term colonic fermentations (i.e. fermentates) were collected at 48 h and used in the co-culture experiments. Caco-2/immune cell challenge assays were executed as previously described^[22]^. Caco-2 cells were maintained in Dulbecco’s Modified Eagle Medium (DMEM) supplemented with 20% heat-inactivated fetal bovine serum (FBS) and 10 mM HEPES. Cells were seeded onto semi-permeable inserts (cellQart) and cultured for 14 days to allow formation of a differentiated monolayer. Monolayer integrity was confirmed at the start of co-cultures by determining transepithelial electrical resistance (TEER; Millicell ERS-2), reflecting barrier function across the epithelial layer. At the start of co-culturing, PWM-stimulated PBMCs were placed in the basolateral compartment, while filter-sterilized (0.22 μm) fermentates from control and chRG-I fermentations were added to the apical side of the Caco-2 monolayer. After 48 h of incubation, barrier integrity was reassessed by TEER measurement. Supernatants from the basolateral compartment were collected for cytokine analysis. Interleukin-6 (IL6), tumor necrosis factor alpha (TNF), and C-X-C motif chemokine ligand-10 (CXCL10) were measured at 24 h, while interferon gamma (IFNG), IL17A, IL4, IL9, IL21, and IL22 were assessed at 48 h. Blank medium from control colonic batch fermentations and supernatant/medium from control Caco-2/PBMC co-cultures were included as controls. All TEER values were normalized to their respective baseline values after subtraction of the empty insert resistance and expressed as percentage of the initial value (set at 100%). Experiments were performed in technical triplicate using PBMCs from one donor. To account for potential biological variability in responses related to PBMCs, similar experiments to those described above were performed with fermented chRG-I samples from a subset of five donors (out of fifteen) using PBMCs from two additional donors and yielded consistent qualitative outcomes.

### Statistical analyses

#### Dietary intervention study:

The statistical framework and analytical workflow followed the same approach as previously described^[13]^. In brief, sample size calculations were based on prior findings from the cRG-I intervention^[14]^, which guided recruitment targets to ensure adequate power for detecting bifidogenic effects. Continuous outcomes were analyzed using Generalized Estimating Equations (GEE) with a Gaussian model to evaluate time and treatment effects, including relevant covariates (such as baseline value, age, sex, BMI, and fiber intake). Binary outcomes were assessed through logistic regression, and model fit was tested via Wald and likelihood ratio chi-squared statistics.

Baseline differences between groups were evaluated using Mann-Whitney *U* or chi-squared tests, and within-group changes were assessed using Wilcoxon signed-rank tests. Analyses were conducted on both the intention-to-treat (ITT) and per-protocol (PP) populations, with comparable results unless otherwise stated. Outlier detection (Grubbs’ test) identified no data exclusions. Statistical significance was set at *P* < 0.05 (two-sided). All analyses were performed using Stata version 12 (StataCorp, College Station, TX, USA) and GraphPad Prism version 6 (GraphPad Software, San Diego, CA, USA).

#### Pre-clinical study:

To evaluate batch fermentation results, paired two-tailed *t*-tests were used to compare the treatment to the blank control across the various donors using per-donor measurements as replicate values. Differences were considered statistically significant if *P* < 0.05. To evaluate differences in TEER at the level of the individual donors, treatments were compared to the negative control using a two-way analysis of variance (ANOVA) with Šídák’s multiple comparisons test. To determine differences in immune markers across donors, each value was normalized to the average of the respective negative control. Then, the average and standard deviation of all colonic donors was calculated on these normalized values. Statistically significant differences compared to the negative control were assessed with a paired, two-tailed t-test using the individual donors as replicates (*n* = 15). All statistics were performed using GraphPad Prism version 10.6.1 for Windows (GraphPad Software, San Diego, CA, USA).

## RESULTS

### Human dietary intervention - study subjects

Of the 257 individuals invited to participate, 116 were screened, and ultimately 55 met the eligibility criteria and were enrolled between September 2023 and March 2024. Participants were randomly assigned to either the placebo group (*n* = 28) or the chRG-I supplementation group (*n* = 27). Among the 55 randomized participants, two assigned to the chRG-I arm discontinued participation due to initiation of antibiotic therapy for reasons unrelated to the trial. As antibiotic use constituted an exclusion criterion, both participants were withdrawn from the study; one at baseline and the other after two weeks of supplementation.

The baseline characteristics of participants are summarized in Table 2. Both intervention arms displayed comparable demographic and anthropometric features. The mean age and sex distribution were closely matched between groups (placebo: 48.4 ± 12.1 years, 67.9% female; chRG-I: 46.2 ± 17.5 years, 69.2% female), with body mass index values indicative of a slightly overweight cohort (placebo: 26.20 ± 3.45; chRG-I: 24.96 ± 4.15 kg*m^-2^) [Table 2 and Supplementary Table 1]. No significant between-group differences were observed for total energy intake or baseline *Bifidobacterium* counts.

**Table 2:** Subjects’ baseline characteristics

**Parameter**	**Statistic**	**Placebo**	**chRG-I**	***P*-value**
N		28	27	
Age (y)	Median (Q1, Q3)	50.50 (42.25, 56.50)	50.00 (28.00, 64.00)	*P* = 0.879
Female sex	N (%)	19 (67.9 %)	19 (69.2 %)	*P* = 0.840
BMI (kg*m^-2^)	Median (Q1, Q3)	26.71 (22.88, 28.20)	23.89 (21.22, 27.96)	*P* = 0.191
*Bifidobacterium* spp. counts^a^ at baseline	Median (Q1, Q3)	0.82 (0.67, 1.14)	1.06 (0.70, 1.22)	*P* = 0.436
Energy (kcal/day)	Median (Q1, Q3)	1817.72 (1535.58, 21.89.66)	1,758.00 (1,587.20, 2,617.06)	*P* = 0.742
Protein (%/day)	Median (Q1, Q3)	18.46 (16.60, 20.37)	16.50 (13.88, 18.92)	*P* = 0.007*
Carbohydrates (%/day)	Median (Q1, Q3)	40.78 (32.13, 45.01)	43.59 (38.69, 48.59)	*P* = 0.033*
Fiber intake (%/day)	Median (Q1, Q3)	2.41 (2.10, 2.92)	2.13 (1.87, 2.70)	*P* = 0.213
Fiber intake (g/day)	Median (Q1, Q3)	23.44 (16.61, 32.25)	23.50 (13.75, 31.99)	*P* = 0.946
Fat (%/day)	Median (Q1, Q3)	37.39 (33.37, 44.19)	37.00 (32.31, 41.32)	*P* = 0.359
Dietary supplements consumption	N (%)	8 (28.6 %)	3 (11.1 %)	*P* = 0.058

^a^Expressed in log *Bifidobacterium* copies/µL, normalized to 100 ng/ µL DNA. Statistical analyses performed with Mann-Whitney *U* test or Chi-square. *: statistically significant compared to placebo (*P* < 0.05). chRG-I: RG-I derived from chicory root.

At baseline, participants in the chRG-I group exhibited a modestly lower contribution of protein to total daily energy intake (16.32 ± 2.57%, *P* = 0.007) and a correspondingly higher proportion from carbohydrates (43.38 ± 6.18%, *P* = 0.033) relative to the placebo group (18.62 ± 2.62% and 39.08 ± 7.49%, respectively). Fiber consumption, however, did not differ significantly between groups. Sub-groups were stratified according to baseline *Bifidobacterium* numbers and habitual fiber intake, as described previously [Supplementary Figure 1]. Adherence to the intervention was high across both groups (placebo: 95.7%; chRG-I: 92.5%), confirming robust compliance and an effective randomization and stratification process.

Furthermore, both interventions were well tolerated, across the 4-week administration period, no treatment-emergent adverse events were recorded, and no withdrawals were attributed to product-related reactions in either the placebo or chRG-I arms.

### Impact of chRG-I intake on *Bifidobacterium* spp. counts

**Figure 2:**
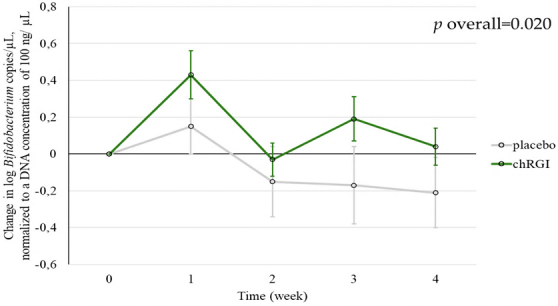
The change in the *Bifidobacterium* spp. counts during 4 weeks after the start of supplementation. Each symbol depicts the mean ± SEM of at least 24 observations. Changes over time were analyzed using GEE with a Gaussian model, including baseline value, age, sex, BMI, and fiber intake as covariates. A statistically significant difference between the chRG-I and placebo groups was observed. chRG-I: RG-I derived from chicory root; SEM: standard error of the mean; GEE: Generalized Estimating Equations; BMI: body mass index.

Participants received a daily supplementation of 500 mg chRG-I or placebo for 4 weeks, during which *Bifidobacterium* counts were assessed at weekly intervals. Both groups (placebo and chRG-I) reported comparable habitual dietary fiber intake (approximately 25 g/day). The chRG-I group exhibited a progressive increase in *Bifidobacterium* levels, while the placebo group displayed a gradual decline across the intervention period [Figure 2]. A marked elevation was evident in the chRG-I group during the first week, followed by a transient reduction at week 2 and a secondary rise at week 3, when the divergence between groups became most pronounced. By week 4, *Bifidobacterium* levels in the chRG-I group had approached baseline yet remained higher than those observed in the placebo arm. Longitudinal analysis using GEE confirmed a significant difference in temporal response between treatments (*P* = 0.020), and individual response distributions are shown in Supplementary Figures 2 and 3. Overall, daily supplementation with chRG-I promoted an early and transient enrichment of *Bifidobacterium* spp., with a clear distinction from placebo particularly evident in the third week of the intervention.

Meanwhile, at all assessed time points, a higher proportion of participants in the group taking chRG-I exhibited enhanced *Bifidobacterium* counts compared with the placebo group. The percentage of people with increased bifidobacteria counts declined between weeks 1 and 2 in both groups; however, while this reduction continued in the placebo group over the following weeks, a transient increase was seen in the chRG-I group between weeks 2 and 3, followed by a modest decline at week 4 [Supplementary Figure 4]. By the end of the intervention, 43% of participants in the placebo group and 52% in the chRG-I group displayed elevated *Bifidobacterium* levels relative to baseline.

### Impact of chRG-I supplementation on microbially derived metabolite profiles

**Figure 3:**
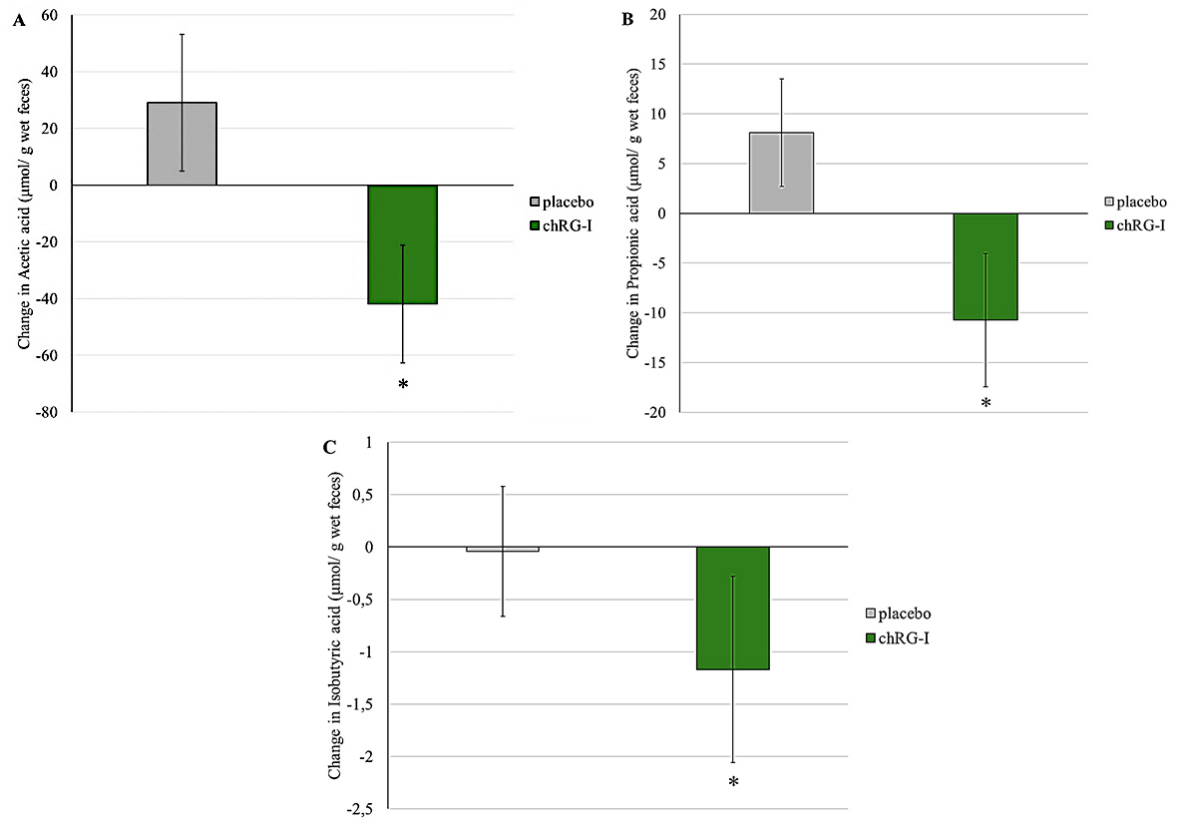
Changes in the concentration of various SCFA; acetic acid (A), propionic acid (B), and isobutyric acid (C) in feces between baseline and week 4 of supplementation. Figures depict the mean ± SEM of at least 22 observations. Changes over time were analyzed using GEE with a Gaussian model, including baseline value, age, sex, BMI, and fiber intake as covariates.*: statistically significant difference compared to placebo (*P* < 0.05). chRG-I: RG-I derived from chicory root; SCFA: short chain fatty acids; SEM: standard error of the mean; GEE: Generalized Estimating Equations; BMI: body mass index.

Supplementation with chRG-I for 4 weeks resulted in slightly lower fecal concentrations of some SCFA compared with placebo [Figure 3A-C]. For acetate, mean concentrations increased from 228.97 ± 169.30 to 258.18 ± 171.28 µmol/g in the placebo group, whereas they decreased from 206.21 ± 143.47 to 163.06 ± 103.41 µmol/g in the chRG-I group (*P* < 0.0001). A similar pattern was observed for propionate, which increased from 49.20 ± 54.99 to 57.10 ± 55.92 µmol/g under placebo and decreased from 52.11 ± 51.45 to 40.32 ± 42.54 µmol/g following chRG-I supplementation (*P* = 0.001). For isobutyric acid, values were stable in the placebo group (7.54 ± 2.68 to 6.93 ± 2.08 µmol/g) but decreased slightly in the chRG-I group (6.53 ± 1.87 to 5.98 ± 1.19 µmol/g), resulting in a statistically significant between-group difference (*P* = 0.043). After 4 weeks, no differences were detected in the concentrations of butyric acid, valeric acid and isovaleric acid in fecal samples [Supplementary Table 2].

### Impact of chRG-I on physiological and well-being indicators

**Figure 4:**
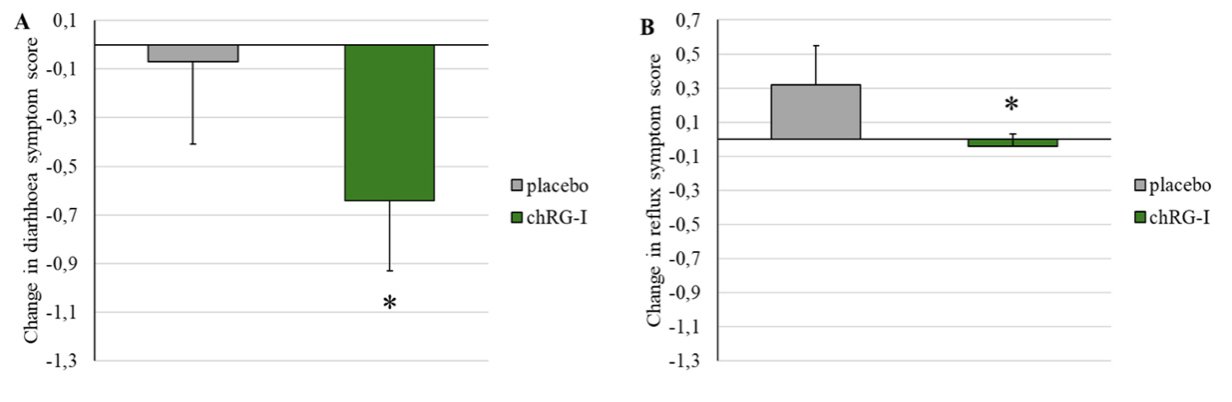
Changes in symptom scores between baseline and week 4 for (A) diarrhea and (B) reflux in healthy subjects receiving placebo or chRG-I. Data are presented as mean ± SEM. Changes over time were analyzed using GEE with a Gaussian model, including baseline value, age, sex, BMI, and fiber intake as covariates.*: statistically significant difference compared to placebo (*P* < 0.05). chRG-I: RG-I derived from chicory root; SEM: standard error of the mean; GEE: Generalized Estimating Equations; BMI: body mass index.

Potential effects on gastrointestinal symptomatology were explored at baseline and following 4 weeks of supplementation [Supplementary Table 3]. Participants receiving chRG-I exhibited a greater reduction in diarrhea-related symptom scores (baseline: 4.35 ± 2.08; week 4: 3.72 ± 1.59) compared with those receiving placebo (baseline: 3.93 ± 1.46; week 4: 3.86 ± 1.58) (*P* < 0.006) [Figure 4A and B]. Among individuals supplemented with chRG-I, 10 of 25 reported a decrease in diarrhea scores by week 4, compared with 7 of 28 in the placebo group. Reflux-associated symptom scores remained largely stable in the chRG-I group (baseline: 2.08 ± 0.27; week 4: 2.04 ± 0.20), whereas a slight increase was observed in the placebo group (baseline: 2.29 ± 0.66; week 4: 2.61 ± 1.29). Despite small absolute differences, the GEE model indicated a statistically significant between-group effect over time (*P* < 0.04). As all participants remained clinically healthy throughout the intervention, the observed reduction in diarrhea scores reflects improved stool consistency rather than the resolution of pathological diarrheal manifestations (maximum score = 7).

No significant between-group differences were detected for other evaluated outcomes, including indices of physical activity [Supplementary Table 4] or self-perceived health status [Supplementary Table 5], and F_cal concentrations remained unchanged.

### Effects of chRG-I on expression of immune cell activation markers

**Figure 5:**
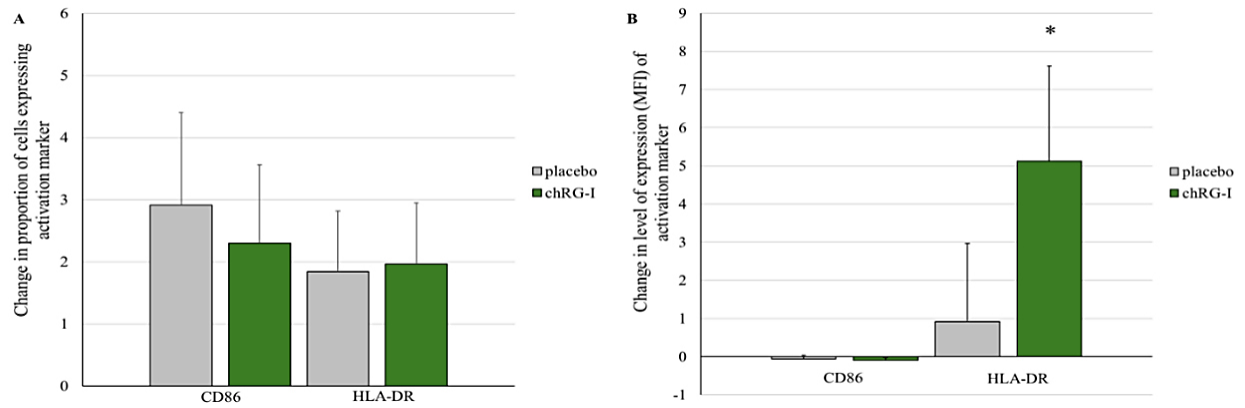
(A) Change in the proportion of myeloid dendritic cells (mDCs) expressing activation markers CD86 and HLA-DR. Expression level (MFI) of activation markers (CD86 and HLA-DR) on mDC after 4 weeks of supplementation with placebo or chRG-I. Data are baseline-corrected and presented as mean ± SEM (%) at week 4. Changes over time were analyzed using GEE with a Gaussian model, including baseline value, age, sex, BMI, and fiber intake as covariates. *: statistically significant difference compared to placebo over time (*P* < 0.05). chRG-I: RG-I derived from chicory root; HLA-D MFI: mean fluorescence intensity; SEM: standard error of the mean; GEE: Generalized Estimating Equations; BMI: body mass index.

No differences were observed in the expression of activation markers on pDCs or macrophages between the treatment groups after 4 weeks of supplementation. Similarly, the proportion of mDCs [Figure 5] expressing CD86 and HLA-DR remained comparable between groups. However, a subtle but statistically significant increase in HLA-DR MFI was detected in the group taking chRG-I compared to the placebo group (*P* = 0.005; Figure 5B), while CD86 MFI remained unchanged.

### Effects of chRG-I on fecal fermentation and epithelial-immune responses *in vitro*

**Figure 6:**
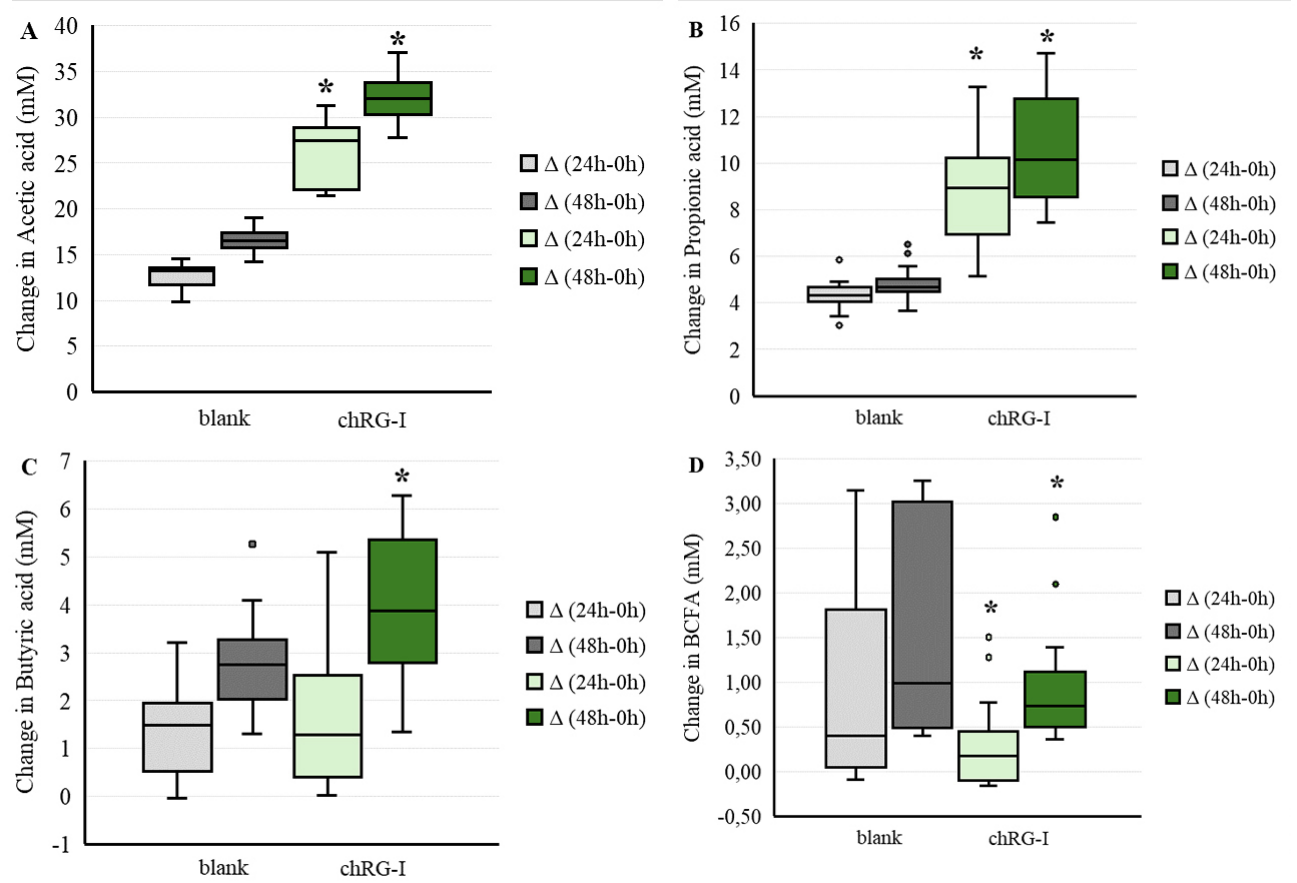
Changes in SCFA and BCFA production during *in vitro* fecal fermentation of chRG-I using fecal samples collected from 15 participants of the intervention study. Fecal inocula were incubated anaerobically with or without chRG-I (blank control). Concentrations of acetate (A), propionate (B), butyrate (C), and total BCFA (D) were determined at 24 h and 48 h and are presented as boxplots showing median, interquartile range, minimum-maximum values and outliers donor values. Differences between chRG-I and blank control were analyzed using paired two-tailed *t*-tests. *: statistically significant differences compared with the corresponding blank control (*P* < 0.05). chRG-I: RG-I derived from chicory root; SCFA: short-chain fatty acids; BCFA: branched-chain fatty acids.

*In vitro* fermentation of chRG-I with fecal microbiota derived from 15 study participants at t = 0 h (prior to the intervention with chRG-I) resulted in pronounced modulation of SCFA and BCFA production over time [Figure 6]. At both 24 h and 48 h of incubation, acetate concentrations were significantly higher in chRG-I fermentations than in the negative control, corresponding to increases of 109% (*P* < 0.0001) and 96% (*P* < 0.0001), respectively [Figure 6A]. Similarly, propionate concentrations in the chRG-I condition exceeded those of the negative control by 105% at 24 h (*P* < 0.0001) and 118% at 48 h (*P* < 0.0001) [Figure 6B]. Butyrate production was also higher following chRG-I fermentation, with concentrations at 48 h exceeding those observed in the negative control by 42% (*P* = 0.0011), while only minimal changes were detected in the blank condition [Figure 6C]. Total BCFA concentrations increased over time; however, levels remained substantially lower with chRG-I than those observed in the blank control, with concentrations approximately 60% lower at 24 h (*P* = 0.0213) and 42% lower at 48 h (*P* = 0.0092) [Figure 6D]. Overall, chRG-I fermentation resulted in a time-dependent increase in SCFA concentrations and consistently lower BCFA levels relative to the negative control.

During *vitro* fermentation, pH decreased over time in the presence of chRG-I, from 6.48 (±0.02) at baseline to 6.02 (±0.05) at the end of incubation across donors, whereas pH values in the blank control remained higher, i.e. 6.51 (±0.02) at 0 h to 6.45 (±0.02) at 48 h [Supplementary Figure 5]. All measured pH values during fermentation were within the physiological range reported for the healthy adult colon (approximately 5.6-6.9).

**Figure 7:**
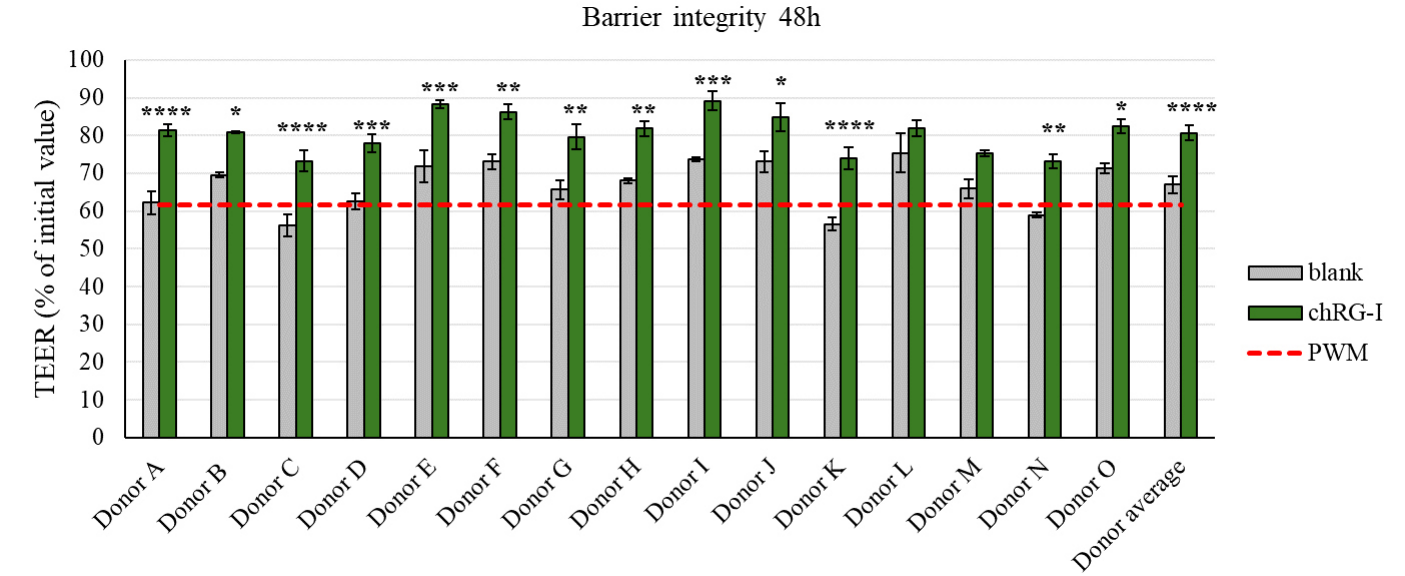
Effect of *in vitro* fermentation supernatants from chRG-I on intestinal barrier integrity after 48 h. The apical side of Caco-2/immune cell co-cultures were exposed for 48 h to fermentation supernatants obtained from 15 donors who participated in the chRG-I intervention study. TEER was measured to assess barrier integrity and is expressed as a percentage of the initial baseline value, which was set at 100%. Grey bars are controls and green bars are chRG-I treatments. Data represent mean ± SD. Red dashed line indicates positive control stimulation with PWM. Differences between treatments and the corresponding blank control were analyzed using two-way ANOVA with Šídák’s multiple comparisons test. * *P* < 0.05, ** *P* < 0.01, *** *P* < 0.001, **** *P* < 0.0001. *P*-values include < 0.0001, 0.0251, < 0.0001, 0.0005, 0.0002, 0.0044, 0.0021, 0.0025, 0.0005, 0.0150, < 0.0001, 0.6048, 0.1242, 0.0017, 0.0291, and < 0.0001 for donors A-O and the average donor, respectively. TEER: Transepithelial electrical resistance; PWM: pokeweed mitogen; chRG-I: RG-I derived from chicory root; SD: standard deviation; ANOVA: analysis of variance.

To evaluate the impact of *in vitro* chRG-I fermentates on epithelial barrier integrity, TEER values of Caco-2 monolayers were recorded before (t = 0 h) and after 48 h exposure. chRG-I-fermentates protected barrier integrity (in 13 out of the 15 samples, green bars, Figure 7) as compared to the blank control fermentates (grey bars, Figure 7) following PMW challenge (*P* < 0.0001).

**Figure 8:**
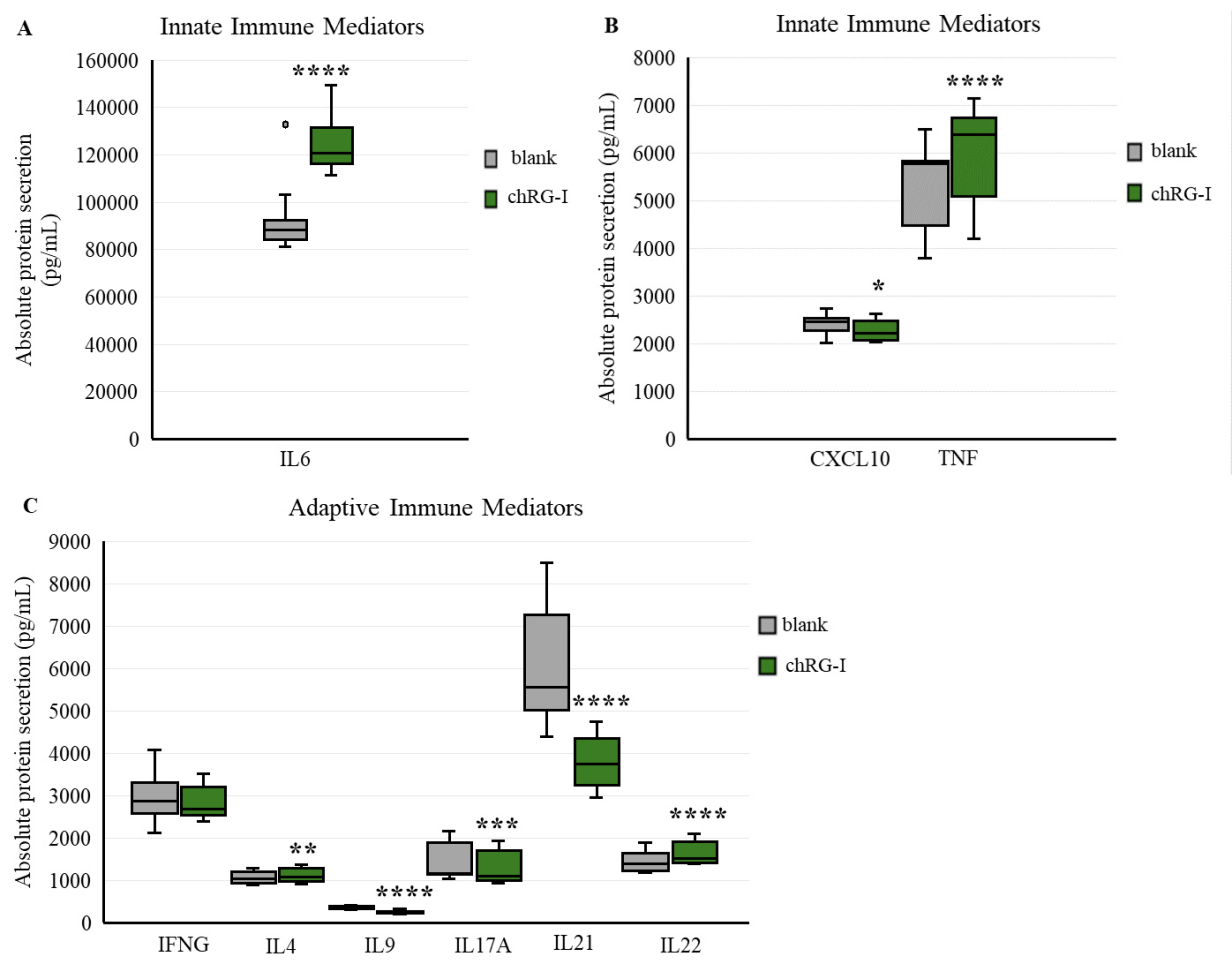
Cytokine release following exposure to in vitro fermentation supernatants of chRG-I. Innate immune mediators IL6 (A), CXCL10 and TNF (B) were quantified after 24 h of exposure, while adaptive immune mediators IFNG, IL4, IL9, IL17A, IL21, and IL22 (C) were measured after 48 h. Data are presented as boxplots showing median, interquartile range and minimum-maximum values of absolute protein secretion (pg/mL) from PBMCs of 15 subjects. Blank fermentation supernatants served as the negative control. Data were normalized to the corresponding negative control per donor, and differences were analyzed using paired two-tailed *t*-tests (*n* = 15). Statistical significance compared with the corresponding blank control: * *P* < 0.05, ** *P* < 0.01, *** *P* < 0.001, **** *P* < 0.0001. chRG-I: RG-I derived from chicory root; *IL*: interleukin; *CXCL*: C-X-C motif chemokine ligand; *TNF*: tumor necrosis factor alpha; *IFNG*: interferon gamma; PBMC: peripheral blood mononuclear cell.

In the co-culture model, exposure of Caco-2 cells (apical compartment) to fermentation supernatants obtained after *in vitro* fermentation of chRG-I led to distinct alterations in cytokine release in the basolateral compartment compared with the blank control [Figure 8A-C]. After 24 h, IL6 and TNF concentrations were significantly increased following incubation with chRG-I fermentates (*P* < 0.0001 for both), whereas CXCL10 levels were reduced (*P* = 0.0273). At 48 h, IL4 and IL22 release was elevated (*P =* 0.0013 and *P* < 0.0001, respectively), while IL9, IL17A, and IL21 levels were decreased relative to the control (*P* < 0.0001, *P* = 0.0019 and *P* < 0.0001, respectively). IFNG secretion remained unchanged upon treatment, compared to the control (*P* = 0.0824). Overall, exposure to chRG-I-fermentates elicited a differential cytokine secretion profile by PBMCs challenged with PWM, which was characterized by the selective modulation of both pro- and anti-inflammatory mediators.

## DISCUSSION

This paper presents the first randomized, double-blind, placebo-controlled clinical study in healthy adults with a novel variant of RG-I, derived from chicory (chRG-I), and provides clinical evidence that supplementation with chRG-I can beneficially modulate the gut microbiota, and can modulate the activation status of immune cells in the systemic compartment. Furthermore, preclinical evidence demonstrates that chRG-I can protect gut barrier integrity during a pro-inflammatory challenge and affect immune signaling. Through the integration of data from a clinical intervention with data from donor-matched *in vitro* fermentation and epithelial assays, this work delineates a coherent biofunctional profile for this previously not studied RG-I source and offers insight into how complex pectic polysaccharides engage with the human host.

We observed that supplementation with a low daily dose of chRG-I (500 mg/day) for four weeks resulted in a statistically significant bifidogenic effect in healthy individuals, even with a relatively high fiber background diet. The increase in absolute *Bifidobacterium* spp. counts peaked around week 3 and remained above placebo through week 4. Transient fluctuations were observed in both groups (including a brief increase in the placebo group at week 1), which likely reflect normal temporal variability in microbiota composition rather than a treatment effect, particularly as the placebo does not contain fermentable fiber. Although the magnitude was modest, this pattern aligns with earlier studies using RG-I (derived from carrots), which reported similar taxa-specific responses^[13,14]^. The consistency of bifidogenic responses across both sources supports the classification of RG-I as a selective saccharolytic substrate with potential prebiotic activity. RG-I thus operates within the general framework of fermentable fibers, while its structural complexity and low-dose efficacy suggest a more selective microbiota-mediated mode of action.

Participants in the chRG-I group exhibited lower fecal concentrations of some SCFA and isobutyrate at week 4 compared with placebo. While this may appear counter-intuitive in light of prior *in vitro* fermentation data showing enhanced saccharolytic activity, this paradox is consistent with the broader literature indicating that fecal SCFA concentrations are weak proxies for colonic SCFA production because most SCFA are rapidly absorbed or utilized by host colonocytes, as well as further redistributed through cross-feeding interactions within the microbial community^[23,24]^. Fecal SCFA levels may be influenced by multiple host- and diet-related factors, including habitual fiber and protein intake, gastrointestinal transit, and overall microbiota functionality. However, in the present study, participants in both groups reported comparable dietary fiber intake at baseline (~ 25 g/day) and were instructed to maintain their habitual diet throughout the intervention. No substantial changes in dietary patterns, body weight, or health status were reported, suggesting a relatively stable physiological and microbial background. Although statistically significant differences in protein and total carbohydrate intake were observed at baseline, the absolute differences were modest (approximately 2%-4%) and remained within ranges consistent with a balanced diet. Importantly, total dietary fiber intake, being the primary substrate for colonic fermentation, was comparable between groups, suggesting that these baseline differences were unlikely to have materially influenced fermentation outcomes.

In contrast, *in vitro* fermentation of chRG-I with donor fecal inocula (donated by subjects taking part in the clinical intervention at baseline) revealed robust generation of acetate, propionate, and butyrate, together with reduced levels of BCFA and pronounced acidification. This difference highlights the point that fecal SCFA measurements represent the net outcome of microbial production, host absorption, and utilization, whereas *in vitro* fermentation captures microbial production in isolation without host-mediated uptake. Within this controlled *in vitro* context, these changes reflect a predominantly saccharolytic rather than proteolytic fermentation profile, as expected and reported previously for this family of complex polysaccharide substrates^[25,26]^. This pattern may contribute to the observed improvement in stool consistency, which, in a healthy cohort, are best interpreted as enhanced gastrointestinal comfort rather than therapeutic effects.

The *in vitro* fermentation and gut barrier model findings provide insights into how chRG-I-derived metabolites may contribute to intestinal barrier protection and local immune regulation. Incubation with chRG-I fermentates protected epithelial barrier integrity in Caco-2/PBMC co-cultures challenged with PWM by preserving TEER as compared to blank control fermentates. In addition, the cytokine profile underwent a distinct qualitative shift upon exposure to these fermentates; we observed a transition away from a potentially damaging pro-inflammatory response, observed in the control, towards a profile associated with epithelial maintenance and mucosal tolerance following chRG-I supplementation. As this model employs PWM-stimulated PBMCs, cytokine secretion reflects an activated, context-dependent response rather than physiological cytokine levels; importantly, these *in vitro* shifts were consistent across multiple donors, supporting a reproducible and regulated modulation rather than excessive immune activation. Similar barrier-protective actions have been documented for RG-I-rich pectins from Goji berry and raspberry where the pectins were administered in their native, non-fermented form, which attenuated cytokine overproduction in colitis models and upregulated tight-junction proteins^[27]^.

Complementing these *in vitro* observations, the *in vivo* immune profiling revealed a mild but consistent systemic response. While earlier dietary intervention studies with RG-I utilized functional assays such as natural killer (NK) cell activity^[28]^ and phagocytic activity^[11]^, this study prioritized high-resolution flow cytometry (FACS) for detecting nuanced shifts in systemic “immune alertness” via the expression of cell surface activation markers. Although NK and phagocytosis assays provide valuable functional snapshots, they are notoriously sensitive to *ex vivo* handling and subject to high inter-assay variability. The small yet measurable increase in HLA-DR expression suggests a subtle enhancement of antigen-presenting capacity and potentially enabling CD4+ T helper cell responses. Such cross-talk between microbial fermentation products and host immune function has been recognized as a defining feature of complex dietary polysaccharides acting through microbiota-dependent metabolic signaling^[29]^.

Through the integration of a randomized controlled human intervention with donor-matched *in vitro* fermentation and gut barrier assays, this study offers some mechanistic insights into how RG-I engages with the human host. These findings also build on earlier observations with cRG-I^[13]^. The botanical origin and differences in fine structural attributes of RG-I variants may shape specific microbiome-host responses, and subtle disparities in responses between chRG-I and cRG-I likely stem from differences in side-chain branching, molecular weight distribution, and degree of acetylation. These factors have been described to influence RG-I structure-function relationships^[30]^.

This study has several strengths, including its randomized, placebo-controlled design and the stratification of participants according to habitual dietary fiber intake and baseline bifidobacterial counts, allowing a more balanced evaluation of intervention effects across distinct microbial and dietary backgrounds. The longitudinal fecal sample collection (every week for four weeks) and predefined statistical method of analysis (GEE) enabled us to perform a fully powered placebo-controlled study with relatively small group sizes within limited resource constraints. The integration of clinical outcomes with matched donor-derived *in vitro* fermentation and intestinal barrier models further provided a translational framework linking microbial activity, metabolite production, and host-related responses.

At the same time, certain limitations should be considered. The relatively short duration of the intervention did not enable detection of longer-term adaptations in gut microbiota composition and function, beyond 4 weeks. Also, measurements of immune function i.e., NK cell activity or phagocytosis were not included in this study, since these parameters are most often measured with success after at least 8 weeks dietary intervention. The small sample size, while statistically powered for the primary outcomes, may have limited sensitivity to detect more subtle or inter-individual effects, particularly within immune-related endpoints. The study was not statistically powered to support formal subgroup (low/high dietary fiber intake or *Bifidobacterium* level) analyses, so these analyses were not performed to avoid overinterpretation of data. In addition, the study was conducted in healthy adults, which may limit generalizability with respect to a typical clinical population for whom the intervention may be useful. Also, results in a healthy population may lead to responses with a magnitude that is (much) smaller than what might be observed in individuals with e.g., impaired gut barrier function, gut microbiota dysbiosis or the presence of underlying inflammatory diseases. From a methodological perspective, while the use of fecal fermentation and epithelial co-culture systems supports mechanistic interpretation, these models cannot fully capture the physiological complexity of the *in vivo* intestinal environment. Although dietary intake was assessed and considered in the analysis, residual variability in habitual diet and lifestyle factors cannot be entirely excluded. Finally, while consistent patterns were observed across clinical and preclinical models, the study design does not allow for definitive conclusions regarding causal relationships between microbial changes, metabolite production, and immune modulation. Further studies with longer follow-up, larger cohorts, and more integrative analytical approaches will be important to better define these interactions.

In conclusion, this first human intervention with chRG-I provides clinical and mechanistic evidence that this structurally complex plant polysaccharide can modulate the microbiota- gut barrier-immune axis in healthy adults. These findings lay the groundwork for a better understanding of structure-function relationships between RG-I variants, and open perspectives for developing tailored fiber formulations that optimize microbial metabolism, gut barrier protection, and immune balance to sustain health across diverse populations.

## DECLARATIONS

### Availability of data and materials

The data presented in this study are available on request from the corresponding author due to ethical restrictions and privacy considerations regarding participant data.

### Copyright

© The Author(s) 2026.
