# Supplementary material for: Dietary chicory rhamnogalacturonan-I modulates gut microbiota and immune responses in healthy adults
Source: Microbiome Res Rep. 2026 May 13;5(2):11. doi: 10.20517/mrr.2026.04 (PMC13246491; doi:10.20517/mrr.2026.04)
Supplement: Supplementary file 1 [file mrr-5-2-11-SupplementaryMaterials.pdf]

Surname *et al. Microbiome Res Rep* Year;Volume:Number
DOI: 10.20517/mrr.xxxx.xx

Microbiome Research Reports

Supplementary Materials

Dietary chicory rhamnogalacturonan-I modulates gut microbiota and immune responses in healthy adults

Evangelia N. Kerezoudi[1,2], Sue McKay[3], Seta Kurt[4], Maaike De Kreek[5], Jelle De Medts[6], Lynn Verstrepen[6], Jonas Ghyselinck[6], Lieven Van Meulebroek[6,7], Wim Calame[8], Ruud Albers[3], Annick Mercenier[3], Robert J. Brummer[1,2], Ignacio Rangel[1,2]

[1]School of Medical Sciences, Örebro University, Örebro 70182, Sweden.
[2]Nutrition-Gut-Brain Interactions Research Centre, Food and Health Center, Örebro University, Örebro 70182, Sweden.
[3]NutriLeads BV, Wageningen 6708 WH, The Netherlands.
[4]Department of Clinical Research Laboratory, Faculty of Medicine and Health, School of Medical Sciences, Örebro University, Örebro 70182, Sweden.
[5]Clinical and Experimental Endocrinology, KU Leuven, Leuven 3000, Belgium.
[6]ProDigest BV, Zwijnaarde 9052, Belgium.
[7]Laboratory of Integrative Metabolomics, Department of Translational Physiology, Infectiology and Public Health, Faculty of Veterinary Medicine, Ghent University, Merelbeke 9820, Belgium.
[8]StatistiCal BV, Wassenaar 2241 MN, The Netherlands.

Correspondence to: Dr. Evangelia N. Kerezoudi, School of Medical Sciences, Örebro University, Örebro 70182, Sweden. E-mail: evangelia.kerezoudi@gmail.com

ORCID: Evangelia N. Kerezoudi (0000-0001-8610-342X)

© The Author(s) 2021. Open Access This article is licensed under a Creative Commons Attribution 4.0 International License (https://creativecommons.org/licenses/by/4.0/), which permits unrestricted use, sharing, adaptation, distribution and reproduction in any medium or format, for any purpose, even commercially, as long as you give appropriate credit to the original author(s) and the source, provide a link to the Creative Commons license, and indicate if changes were made.

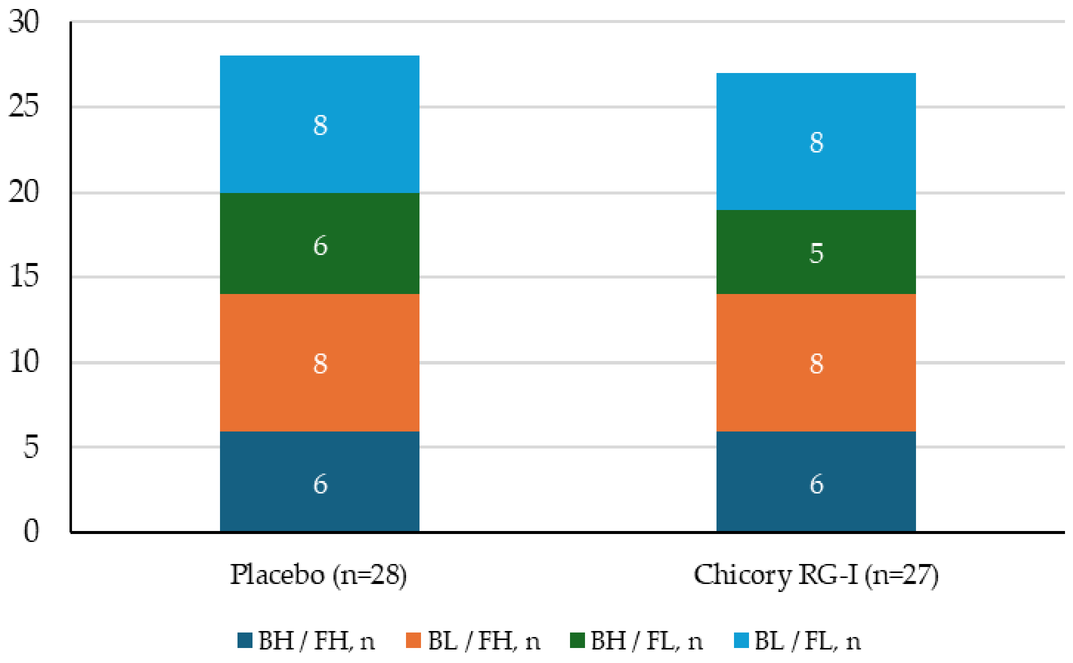

Figure S1. Participants sub-group allocation based on baseline *Bifidobacterium* spp. counts and daily fiber intake. BH/ BL: High or Low *Bifidobacterium* spp. counts at baseline compared to the *Bifidobacterium* spp. levels measured in participants using the median value obtained from group A enrolled in the study (log *Bifidobacterium* copies/µl, normalized for DNA concentration of 100 ng/µl $\geq$ or $<$ 0.894). FH/ FL: High or Low daily dietary fiber intake at baseline compared to the daily dietary fiber intake of participants using the median value obtained from group A enrolled in the study (daily dietary fiber intake $\geq$ or $<$ 22.66 g/day).

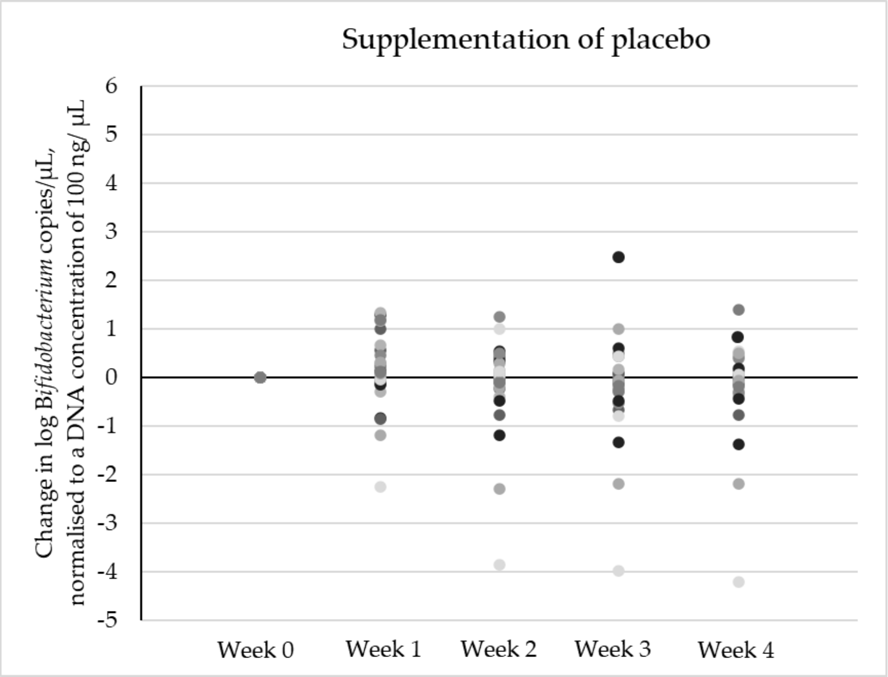

Figure S2. Distribution of individual changes in *Bifidobacterium* spp. counts during

four weeks of placebo supplementation. Each dot represents one subject, and values are expressed as change in log *Bifidobacterium* copies/µL normalized to a DNA concentration of 100 ng/µL.

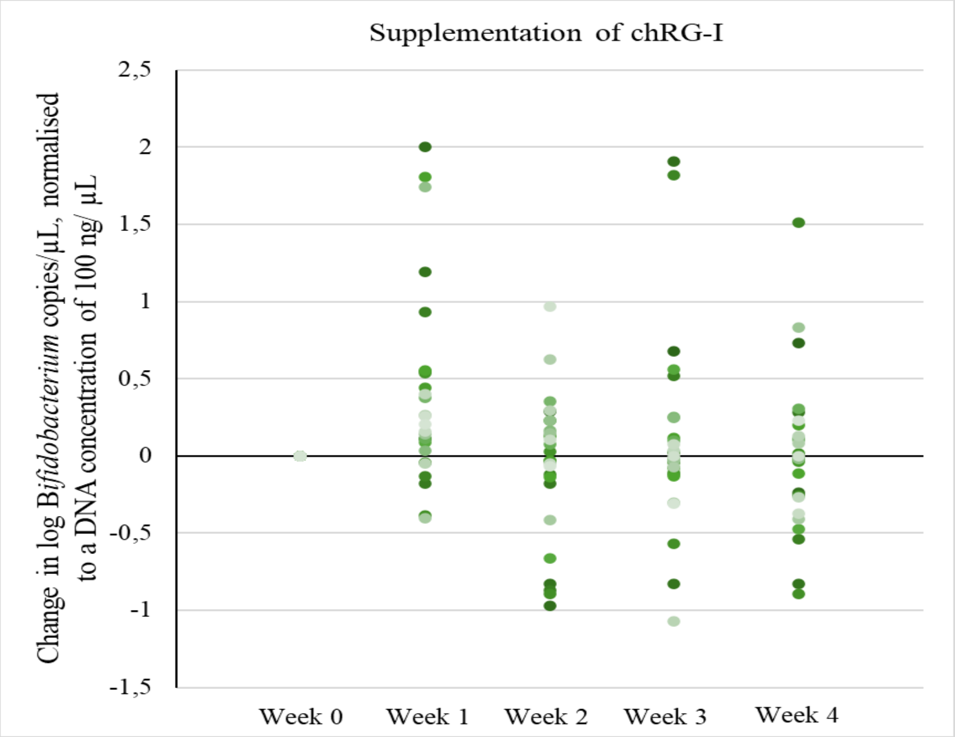

Figure S3. Distribution of individual changes in *Bifidobacterium* spp. counts during four weeks of chRG-I supplementation. Each dot represents one subject, and values are expressed as change in log *Bifidobacterium* copies/µL normalized to a DNA concentration of 100 ng/µL.

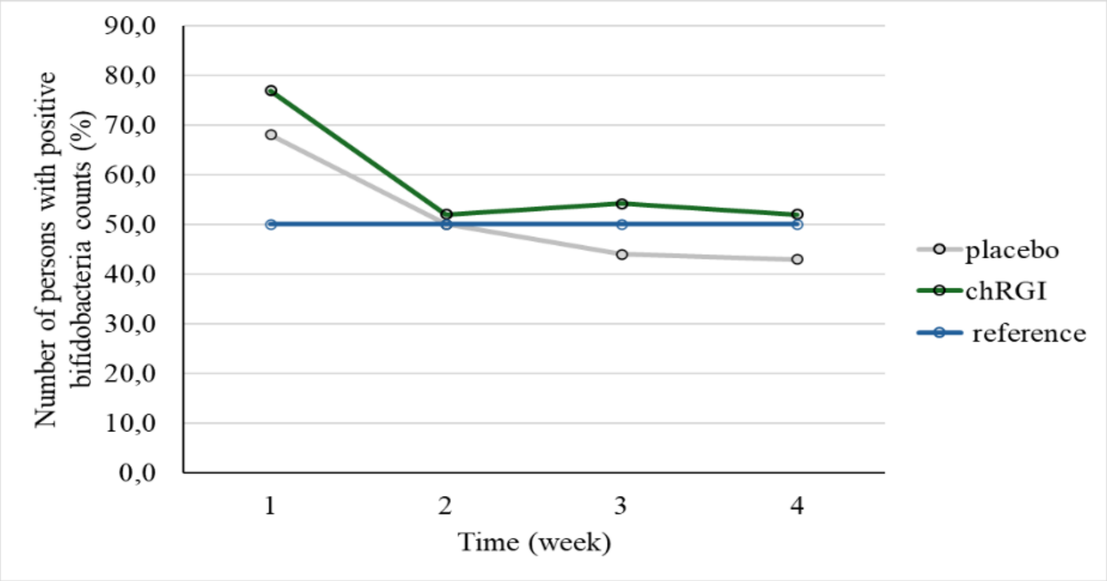

Figure S4. The change in the percentage of persons with an increase in *Bifidobacterium* spp. counts (as compared to the value at the start) during 4 weeks after the start of the consumption of various compounds. Baseline (week 0) was used as the reference point. A person was regarded as "positive" at a given week (week 1–4) if their absolute bifidobacteria counts were higher than at baseline. Each figure point depicts the absolute outcome of at least 24 persons.

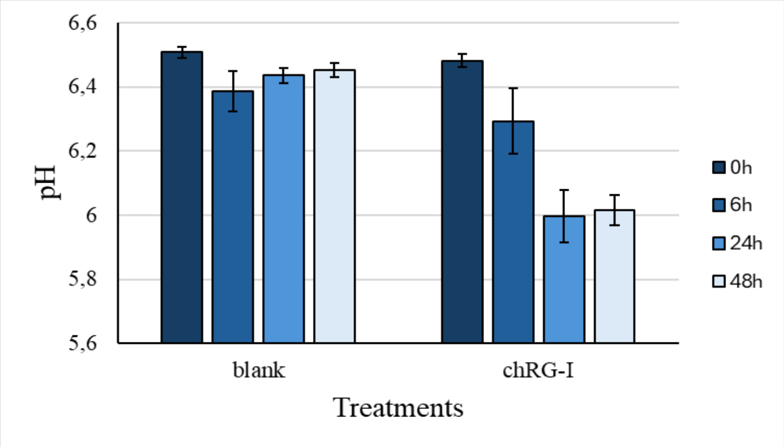

Figure S5. Temporal changes in pH during *in vitro* fecal fermentation of chRG-I. Fecal samples from 15 participants of the intervention study were incubated anaerobically with or without chRG-I (blank control) for up to 48 h. pH values were recorded at 0, 6, 24, and 48 h. Data are presented as mean ± SD.

Table S1. Characteristics of subjects after 4 weeks intervention

Parameter	Placebo	chRG-I	Placebo	chRG-I
	Baseline		Week 4	
BMI (kg*m^{-2})	26.2 ± 3.45	24.96 ± 4.15	26.26 ± 3.38	25.12 ± 4.09
Body weight (kg)	76 ± 10.04	71.59 ± 13.97	76.19 ± 9.85	72.32 ± 13.5

Data is expressed as mean ± SD. BMI: Body Mass Index.

Table S2. Effects of chRG-I dietary supplementation on fecal SCFAs and BCFAs (absolute values (μmol/g feces)

Fecal Microbial Metabolites	Placebo Baseline	Placebo Week 4	chRG-I Baseline	chRG-I Week 4
Acetic acid	228.97±169.30	258.18±171.28	206.21±143.47	163.06±103.41

(N ≥ 25)				
Propionic acid (N ≥ 24)	49.20±54.99	57.10±55.92	52.11±51.45	40.32±42.54
Butyric acid (N ≥ 24)	41.79±28.31	46.59±26.35	45.91±43.77	32.51±25.78
Valeric acid (N ≥ 15)	8.08±2.78	9.03±4.72	8.12±5.56	8.09±3.63
Isobutyric acid (N ≥ 14)	7.54±2.68	6.93±2.08	6.53±1.87	5.98±1.19
Isovaleric acid (N ≥ 16)	8.65±3.51	8.71±3.34	8.93±3.71	8.29±2.48

Data is expressed as mean ± SD.

Table S3. GSRS data at baseline and after 4 weeks intervention

Parameter	Placebo	chRG-I	Placebo	chRG-I
	Baseline		Week 4	
Abdominal pain	4.21 ± 1.95	4.27 ± 1.56	3.86 ± 2.10	3.84 ± 1.65
Reflux	2.29 ± 0.66	2.08 ± 0.27	2.61 ± 1.29	2.04 ± 0.20
Diarrhea	3.93 ± 1.46	4.35 ± 2.08	3.86 ± 1.58	3.72 ± 1.59
Indigestion	6.93 ± 2.54	7.08 ± 2.23	7.25 ± 3.52	7.12 ± 3.05
Constipation	4.96 ± 2.99	4.73 ± 3.23	4.64 ± 2.28	4.60 ± 2.42
Total GI symptoms	22.32 ± 6.45	22.50 ± 5.38	22.21 ± 7.47	21.32 ± 5.80

Data is expressed as mean ± SD.

Table S4. IPAQ-SF data at baseline and after 4 weeks intervention

Parameter	Placebo	chRG-I	Placebo	chRG-I
	Baseline		Week 4	
Total PA (MET-min/week)	2657.89 ± 1988.84	2718.08 ± 2222.85	2704.64 ± 1738.50	2556.28 ± 1796.24
Vigorous PA (MET-min/week)	1001.43 ± 977.34	980.80 ± 1154.01	699.26 ± 895.69	511.67 ± 518.99
Moderate PA (MET-min/week)	645.71 ± 693.99	594.62 ± 657.50	910.71 ± 877.66	561.60 ± 577.84
Walking (MET-min/week)	1010.74 ± 1089.53	1180.38 ± 1590.01	1161.11 ± 1271.69	1503.48 ± 1576.77

Data is expressed as mean ± SD. PA: Physical Activity; MET: Metabolic equivalent of task.

Table S5. EQ-5D-5L data at baseline and after 4 weeks intervention.

Parameter	Placebo	chRG-I	Placebo	chRG-I
	Baseline		Week 4	
Mobility	1.07 ± 0.26	1.04 ± 0.20	1.04 ± 0.19	1.00 ± 00
Self-care	1.04 ± 0.19	1.04 ± 0.20	1.04 ± 0.19	1.04 ± 0.20
Usual activities	1.07 ± 0.26	1.08 ± 0.28	1.07 ± 0.26	1.08 ± 0.28
Pain / discomfort	1.46 ± 0.64	1.44 ± 0.58	1.46 ± 0.58	1.44 ± 0.51
Anxiety/ depression	1.32 ± 0.61	1.44 ± 0.51	1.50 ± 0.79	1.48 ± 0.59
Health perception	78.75 ± 8.20	83.36 ± 8.61	76.61 ± 13.20	79.52 ± 7.29

Data is expressed as mean ± SD.
